# The Role of Sarcopenia in Heart Failure with Depression

**DOI:** 10.31083/j.rcm2309296

**Published:** 2022-09-05

**Authors:** Ruting Wang, Jiahao Duan, Wei Liu, Kai Huang, Zijun Chen, Chun Yang, Ling Yang

**Affiliations:** ^1^Department of Cardiology, The Third Affiliated Hospital of Soochow University, 213003 Changzhou, Jiangsu, China; ^2^Department of Cardiology, Shanghai East Hospital, School of Medicine, Tongji University, 200092 Shanghai, China; ^3^Department of Anesthesiology and Perioperative Medicine, The First Affiliated Hospital of Nanjing Medical University, 210029 Nanjing, Jiangsu, China

**Keywords:** heart failure, depression, comorbidity, sarcopenia, cachexia

## Abstract

Heart failure (HF) and depression are both major medical health issues in our society. Currently, an increasing number of studies demonstrate an association between HF and depression. The prevalence of depression is higher in patients with HF, and depression also increases the incidence of HF. Currently, depression has been listed as a major risk factor for heart disease. Patients with HF and comorbid depression have significantly higher rates of hospitalization and mortality, and clinical symptoms manifest as decreased activity tolerance and decreased muscle mass. Enhancement of the muscle function improves the prognosis of patients with HF and depression. Sarcopenia is defined as age-related loss of skeletal muscle mass plus loss of muscle strength and/or reduced physical performance, and its pathogenesis involves malnutrition, physical inactivity, endocrine disorders and chronic inflammation, which are also involved in the pathogenesis of HF with comorbid depression. Therefore, it would be intriguing to explore the linkage between HF, depression and sarcopenia. This review presents an overview of HF with comorbid depression and sarcopenia, elucidates the mechanisms involved in these disorders, and finally summarizes the treatment strategies of HF with comorbid depression and sarcopenia.

## 1. Introduction

Heart failure (HF) is one of the most common chronic diseases and is the final phase of heart diseases of various etiologies. It has become a major public health problem owing to the high rates of morbidity, mortality and rehospitalization associated with it [1]. 2021 European Society of Cardiology (ESC) guidelines for HF indicate that the prevalence of HF in European adults is approximately 5/1000 person-years [2]. Given the high prevalence of HF in the elderly population (>10% in individuals >75 years old), comorbidities are more common in patients with HF and have a significant impact on their quality of life and long-term prognosis. Depression, a common HF comorbidity, is an independent predictor of major clinical events and death in patients with HF [3]. The prevalence of depression in the global population of patients with HF is 41.9%, with a prevalence of 28.1% for moderate to severe depression [4]. Patients with HF and comorbid depression often present with depressed mood, weight changes, decreased appetite, fatigue and dyspnea; however, most symptoms are not directly caused by impaired cardiac pumping, hemodynamic disorders or psychological factors [5]. Very recently, the pathophysiological mechanisms of skeletal muscle lesions involved in HF with comorbid depression attracted increasing attention. Reduced myocardial contractility, inadequate skeletal muscle perfusion and a systemic low-level inflammatory state in HF results in reduced skeletal muscle mass and function [6]. Furthermore, depressive disorders either trigger or exacerbate a poor lifestyle, reduce exercise tolerance and further exacerbate skeletal muscle pathology, thereby leading to the development of sarcopenia [7].

## 2. Comorbidity of HF and Depression

Depression is mainly manifested as a mental state of a depressed mood and an aversion to activity with a core symptom of anhedonia. HF was highly associated with depressive symptoms in a large population study [8]. Similarly, multiple meta-analyses suggested that patients with HF and comorbid depression had a significantly higher risk of mortality and cardiovascular events with the effect sizes remaining significant after adjustment for confounding factors [9]. Contrarily, depression also appears to increase the risk of HF in patients with no previous history of cardiovascular diseases. The Nord-Trøndelag Health Study (HUNT 2), which included 62,567 healthy patients, found that the severity of depressive symptoms was also highly associated with the incidence of HF after 11 years of follow-up [10]. Thus, HF with comorbid depression has a significant impact on the exacerbation and the mortality of both depression and HF. Currently, both the ESC and the American College of Cardiology/American Heart Association (ACC/AHA) recommend the screening and treatment of depression in patients with HF [1, 2]. However, depression overlaps with HF symptoms, and there are difficulties in the determination of depressive symptoms in patients with HF. Decreased activity tolerance is one of the most common manifestations of both HF and depression, and enhancement of muscle function improves the prognosis of patients with HF and depression [11]. In addition, somatic performance in the depression scale was more strongly associated with all-cause mortality in HF than cognitive performance [12]. The muscle hypothesis suggests that left ventricular dysfunction causes reduced peripheral perfusion and decreased exercise capacity and also triggers skeletal muscle myopathy. The above changes further lead to abnormal sympathetic activation, vasoconstriction, and endothelial dysfunction and ultimately exacerbate the deterioration of left ventricular function [13, 14]. Thus, muscle function may play a significant role in HF combined with depression.

## 3. Alterations in Muscle Function in Patients with HF and Comorbid Depression 

### 3.1 Sarcopenia

Skeletal muscle accounts for 40%–50% of the total body weight and is an important component of the human body [15]. Under physiological conditions, muscle mass declines by an average of 0.47% and 0.37% per year in men and women, respectively. Such a decline can reach 1%–2% per year with aging [16]. In this regard, I. H. Rosenberg coined the term sarcopenia to describe skeletal muscle loss [17]. Sarcopenia refers to a form of muscle atrophy that occurs with aging and is characterized by a degenerative loss of skeletal muscle weight, mass and strength independent of weight gain or loss [18, 19]. Overall, 5%–13% of people aged more than 60 years have sarcopenia, whereas the prevalence in those above 80 years old exceeds 50% [19].

Sarcopenia is defined as low appendicular skeletal muscle mass (ASM), which means that the sum of the muscle mass of the extremities divided by the square of the height is less than two standard deviations from the reference value for young people of the same sex. Asian Working Group for Sarcopenia (AWGS) 2019 consensus defined sarcopenia as age-related loss of skeletal muscle mass plus loss of muscle strength and/or reduced physical performance [20]. In most cases, ASM is measured *via* dual-energy X-ray absorptiometry (DEXA); however, in some cases, bioelectrical resistance can be employed as an alternative. A cutoff value of 7.0 ${\mathrm{kg/m}}^{2}$ for men and 5.4 ${\mathrm{kg/m}}^{2}$ for women for muscle mass measurement *via* DEXA and a cutoff value of 7.0 ${\mathrm{kg/m}}^{2}$ for men and 5.7 ${\mathrm{kg/m}}^{2}$ for women for bioelectrical impedance measurement were suggested by the AWGS. In addition, grip strength (<26 kg for men and <18 kg for women) and general gait speed (<1.0 m/s) should be taken into account [20]. Given that these diagnostic methods are cumbersome and expensive, in recent years researchers have been working to explore biomarkers for the diagnosis and prediction of sarcopenia, such as serum creatinine, type VI collagen turnover-related peptides, and myokines [21].

Sarcopenia is independently associated with several cardiovascular diseases and their associated risk factors, including myocardial infarction, congestive HF, atrial fibrillation, and atherosclerosis [22, 23]. Sarcopenia may accelerate the progression of chronic diseases such as cancer and HF. Indeed, sarcopenia can be found in 20–50% of patients with heart failure with reduced ejection fraction (HFrEF) and is commonly associated with increased morbidity and mortality [2]. The prevalence of sarcopenia in patients with heart failure with preserved ejection fraction (HFpEF) was found to be 19.7% in the SICA-HF study (European multicenter study) [24, 25]. In turn, chronic HF can worsen the adverse outcomes associated with sarcopenia, including osteoporosis, falls, cachexia, frailty, rehospitalization, and death, which may be associated with HF-related malnutrition, altered hormone levels, inflammation, oxidative stress, autophagy, and apoptosis [26]. Bekfani *et al*. [27] have showed recently in skeletal muscle biopsies elevated levels of atrophy genes and proteins in patients with HFpEF compared to HFrEF and healthy controls. Furthermore, patients with HF showed distorted fatty acid oxidation, glucose oxidation and mitochondrial number and function. Patients with reduced muscle function showed elevated levels of inflammatory parameter and reduced fatty acid oxidation.

Very recently, an association between sarcopenia and depression has gradually attracted attention. Depression leads to alterations in neurological, immune, and endocrine functions, which are significantly associated with physical fitness. A meta-analysis including 10 studies revealed that sarcopenia remained significantly positively associated with depression after adjustment for potential confounders, such as age, gender, cognitive performance, and physical activity [28]. In China, a cohort study demonstrated that sarcopenia was also significantly associated with depressive symptoms after adjustment for confounders and that new-onset depressive symptoms were associated with muscle mass, suggesting that sarcopenia is an independent risk factor for depressive symptoms [29].

### 3.2 Cachexia

Unlike sarcopenia, cachexia is defined as complex metabolic syndrome associated with underlying illness and characterized by loss of muscle with or without loss of fat mass. Its major clinical feature is a >5% oedema-free body weight loss during the previous 12 months or less. Cachexia is a generalized wasting process that may occur in 5–15% of patients with HF [2]. In sarcopenia, energy expenditure is not usually increased or it is even decreased, whereas in cachexia, it can be increased due to the hypermetabolic state, and the systemic inflammatory response is more severe in patients with cachexia [14, 30]. Sarcopenia in patients with chronic HF may eventually progress to cardiac cachexia. Previous clinical studies found that cachexia remained an independent predictor of death in patients with HF after adjustment for age, New York Heart Association (NYHA) classification, left ventricular ejection fraction (LVEF), and peak oxygen consumption [31]. Several studies have demonstrated that skeletal muscle is the first tissue to be lost and that the loss of adipose tissue gradually begins later during the course of HF. Thus, patients with HF may experience muscle loss before the onset of cachexia, and muscle loss can lead to increased cachexia [14].

## 4. Mutual Mechanisms

**Fig. 1.**
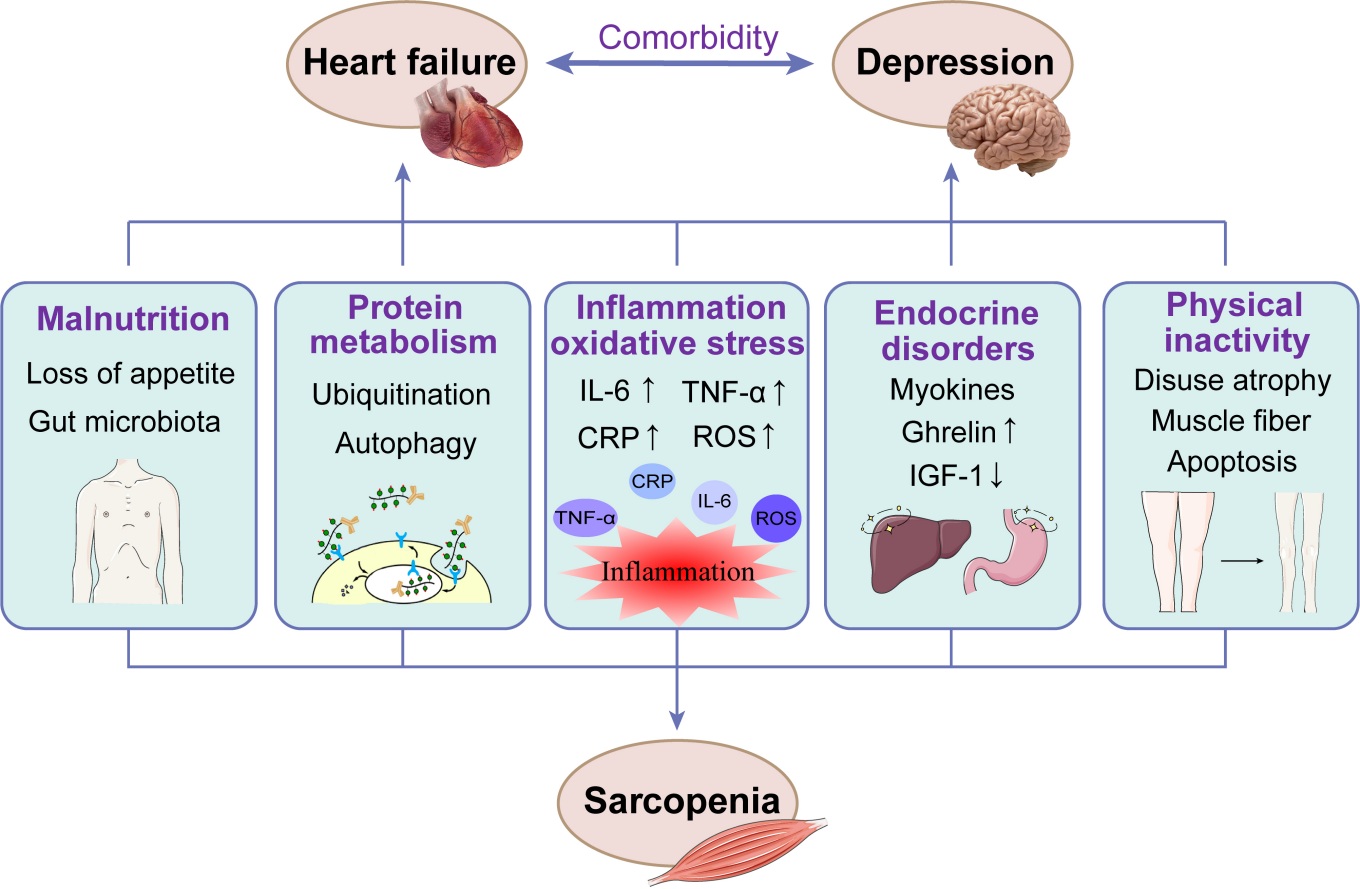
**The mechanisms involved in the pathogenesis of sarcopenia, HF and depression**. CRP, C-reactive protein; IL-6, interleukin 6; IGF-1, Insulin-like growth factor 1; ROS, reactive oxygen species; TNF-α, tumor necrosis factor α.

Recent studies have found that sarcopenia and HF with comorbid depression seem to share some common risk factors, such as malnutrition, chronic inflammation, dysregulation of the hypothalamic–pituitary–adrenal axis (HPA axis), and physical inactivity [5, 32]. The mechanisms involved in the pathogenesis of sarcopenia, HF and depression are presented in Fig. 1.

### 4.1 Malnutrition

A study conducted by Chen *et al*. [33] on the relationship between sarcopenia, depression, and cognitive function demonstrated that subjects with sarcopenia were significantly more malnourished than those without, and there was also a significant difference in nutritional scores between subjects with depressive symptoms. The most direct cause of malnutrition in patients with HF and depression is loss of appetite, which is often associated with symptoms such as dyspnea and nausea but also with side effects of standard drug therapy for HF (such as digoxin, angiotensin-converting enzyme inhibitors, beta-blockers, and diuretics). Patients with severe HF often suffer from intestinal edema associated with loss of taste, nausea, and gastrointestinal disorders, which can lead to loss of appetite and malabsorption [34]. It is worth noting that the gut microbiota, which is known as the “second largest genome”, is widely involved in nutrient absorption, regulating intestinal epithelial function and influencing local or systemic immune inflammatory responses in the intestine; intestinal metabolites can also be released into the blood to play a systemic regulatory role [35]. In addition, blood redistribution and depression in patients with HF can lead to the upregulation of zonulin 2 precursor expression and the downregulation of zonula occludens-1 (ZO-1) expression, resulting in increased intestinal permeability and impaired intestinal epithelial barrier function. This could, in turn, lead to bacterial translocation and endotoxin release into the blood, further aggravating cardiomyocyte and myocyte injury [36]. It is worth noting that loss of appetite is a long-standing problem, and fatigue, depressive symptoms, and low quality of life are independent predictors of appetite decline over time [37].

### 4.2 Protein Metabolism

The proteasome is a protein complex that labels and degrades unwanted or damaged proteins with the help of a small protein called ubiquitin. The entire system of ubiquitination and proteasome degradation is known as the ubiquitin–proteasome system (UPS). Excessive activation of the UPS leads to increased proteolytic metabolism, resulting in an imbalance in myogenic fibronectin levels and causing muscle atrophy [38]. Monoubiquitinated target proteins are degraded by lysosomes, whereas the degradation of target proteins by UPS requires polyubiquitination. E3 ubiquitin–protein ligase is the rate-limiting enzyme for the polyubiquitination of protein substrates, and its most relevant to sarcopenia are tripartite motif-containing 63 (TRIM63) and F-box only protein 32 (FBXO32) [39, 40, 41]. TRIM63 and FBXO32 are mainly regulated by the transcription factors nuclear factor-κB (NF-κB) and forkhead box O (FOXO) protein family [42]. Activated NF-κB and FOXO, in turn, are mainly induced by proinflammatory cytokines, such as tumor necrosis factor (TNF), which is often highly expressed in patients with HF [43].

Autophagy is another important mechanism of sarcopenia in which the lysosomal protease histone L plays a major role. Under normal conditions, autophagy is considered to be a nonselective degradation pathway of unnecessary or nonfunctional cellular components, such as damaged organelles and protein polymers [44]. Thus, autophagy under physiological conditions plays a significant role in maintaining normal muscle function. However, excessive autophagy exacerbates muscle atrophy, and excessive accumulation of autophagic vesicles occurs in almost all myopathies. Autophagy is usually increased through the FOXO and adenosine monophosphate-dependent protein kinase pathways, leading to myofiber atrophy. Studies have demonstrated that both UPS and autophagy are induced in the skeletal muscle early in the course of HF [45]. The above suggests that although autophagy is involved in the maintenance of muscle function, it may be detrimental when induced under catabolic conditions.

### 4.3 Inflammatory Response and Oxidative Stress

HF is a disease characterized by chronic low-level inflammation. Inflammation not only affects cardiovascular function but also has a persistent effect on the skeletal muscle. Furthermore, chronic inflammation is thought to be associated with the pathogenesis of depression, with inflammation and inflammatory diseases causing mood disorders and poor mental health [46]. A cross-sectional study by Visser *et al*. [47] demonstrated that high levels of interleukin 6 (IL-6) and TNF-α in elderly people are associated with lower muscle mass and strength. In addition, a prospective study on inflammation and muscle strength by Schaap *et al*. [48] indicated that the risk of muscle strength loss in elderly people was associated with high levels of IL-6 and C-reactive protein (CRP), suggesting that some inflammatory factors are directly involved in the pathogenesis of sarcopenia. The above inflammatory factors can induce apoptosis, enhance protein hydrolysis and inhibit muscle structural protein gene transcription. Inflammatory cytokines can further activate UPS and induce anorexia and lipolysis, leading to sarcopenia and even cachexia [49]. In addition, HF and depression, which lead to reduced exercise and a sedentary lifestyle, can induce inflammation in the body, releasing inflammatory mediators that destroy the muscle structure and exacerbate fat accumulation, causing the muscle strength to decrease and further promoting reduced activity, thus forming a vicious cycle [50].

The abnormal release of reactive oxygen species (ROS) due to oxidative stress (OS) is a major cause of cellular senescence and apoptosis and often manifests as metabolic abnormalities. OS is extensively involved in diseases associated with aging, including sarcopenia and HF [51]. Studies have demonstrated elevated levels of OS-related markers in patients with HF and an association with reduced exercise tolerance and poor prognosis, which may be related to the high rate of anaerobic metabolism imposed on the organism by a low cardiac output and skeletal muscle hypoxia due to endothelial dysfunction [52]. In addition, excessive ROS can lead to mitochondrial dysfunction, which results in cytochrome c release and activation of caspase 3 and caspase 9, accelerating skeletal muscle injury and degeneration, especially by disrupting the excitation–contraction coupling structure of the muscle [53, 54].

### 4.4 Endocrine Regulation

Cardiac and skeletal myocytes could secrete different myokines that are released into the circulation in an autocrine, paracrine, or endocrine manner to regulate the body’s energy metabolism, insulin sensitivity, lipolysis, free fatty acid oxidation and glycogen metabolism [55]. Myostatin have been shown to be elevated in skeletal muscle in patients with HFpEF or HFrEF [27]. Some of the myokines exert metabolic regulatory effects related to the prevention of cardiovascular diseases, such as myostatin, irisin, apelin, and brain-derived neurotrophic factor (BDNF) [56]. Myostatin, also known as growth differentiation factor 8 (GDF8), is a member of the transforming growth factor β (TGF β) protein family, a class of proteins produced and released by myocytes that inhibit myocyte growth [57]. Studies have demonstrated that myostatin plays a key role in the regulation of skeletal muscle mass and cardiac muscle mass. The activation of the myostatin precursor complex promotes myostatin binding to activin receptor type IIB (ActRIIB) on myofibrils, and downstream mediators cause decapentaplegic homolog 2 (SMAD2) and decapentaplegic homolog 3 (SMAD3) phosphorylation and also affect the corresponding gene transcription [58]. Elevated levels of myostatin have also been observed in HF. Although myostatin may exert some antimyocardial hypertrophic effect, it also promotes myocardial fibrosis [59]. Irisin is produced in large amounts in cardiac and skeletal muscle and protects the myocardium from ischemia-reperfusion injury. Irisin is also involved in central and peripheral neurogenesis [60]. Apelin is associated with the induction of mitochondrial production, which reduces inflammation, stimulates regenerative capacity, and avoids age-related muscle atrophy. Apelin is also involved in physiological processes such as metabolism, cardiac contraction, angiogenesis, and blood pressure regulation [61]. Studies have shown that increased levels of BDNF are detected in human skeletal muscle after exercise. Specifically, tropomyosin receptor kinase B (TrkB) protects against myocardial repair through dimerization with BDNF and intracellular kinase-specific tyrosine phosphorylation, ultimately enhancing the proliferation and survival of cardiac microvascular endothelial cells [56].

Ghrelin expressed in the gastric fundus stimulates the secretion of growth hormone (GH), cortisol, aldosterone, catecholamines and prolactin [62]. The body’s anabolic and catabolic activities are closely related to the aforementioned hormones. For example, ghrelin induces the secretion of GH, which, in turn, exerts anabolic effects directly or indirectly through insulin-like growth factors. Plasma ghrelin levels reflect the nutritional status of the individual and the level of body fat storage. Ghrelin has been found to be significantly increased in patients with cardiac cachexia [63].

Insulin-like growth factor 1 (IGF-1) is a hormone with a molecular structure similar to that of insulin and plays an important anabolic role in the adult body [64]. Contrarily, diminished IGF-1 function is an important component of the sarcopenia process and is not associated with the downregulation of IGF-1 or IGF-1 receptor expression [65]. Mechanistically, upon binding of IGF-1 to its receptor, insulin receptor substrate 1 (IRS1) is activated by phosphorylation and subsequently activates the phosphoinositide 3-kinase (PI3K)-protein kinase B (PKB/AKT)-mammalian target of rapamycin (mTOR) signaling pathway. The PI3K-AKT-mTOR pathway inhibits FOXO and glycogen-synthase kinase 3 (GSK3) and suppresses the activation of SMAD2 by myostatin, leading to increased protein synthesis [66]. Moreover, GH facilitates amino acid delivery to the skeletal muscle and inhibits protein hydrolysis via downstream signaling from IGF-1. However, the number of GH receptors decreases with age, and GH deficiency and lower levels of IGF-1 are associated with an increased risk of endothelial dysfunction and cardiovascular events [67]. In addition, Gold *et al*. [68] found increased secretion of corticotropin-releasing hormone (CRH) in depressed patients, which leads to increased secretion of epinephrine and norepinephrine in the body and induces an inflammatory response. Epinephrine and norepinephrine can lead to endothelial dysfunction, blood flow heterogeneity, and reduced capillary density, which are associated with the development of sarcopenia [69].

### 4.5 Physical Inactivity

Patients with depression have sedentary lifestyles. A retrospective study by Burton *et al*. [70] demonstrated that depressed patients exhibited reduced daytime activity compared with healthy controls. Patients with HF also show a lack of exercise due to physical limitations. From a neurological point of view, physical inactivity also means a reduction in the activity of motor units. Unused or rarely used neurons undergo disuse degeneration, which, in turn, leads to further disuse degeneration of their synaptic junctional cells [71]. Thus, physical inactivity due to disease or sedentary behavior can cause disuse atrophy of the muscles.

In addition, the skeletal muscle is composed of different types of fibers. Among them, type I fibers are rich in mitochondria, muscle protein content and associated capillaries and have low ATPase, creatine kinase and glycolytic activities. Therefore, type I fibers efficiently use oxygen for sustained and slow muscle contraction [72, 73]. Contrarily, type II fibers have high ATPase, creatine kinase and anaerobic glycolytic activity, so type II fibers primarily use anaerobic metabolism for rapid contraction [74]. Muscle fiber types can change in response to external stimulation. Aging-induced loss of peripheral motor neurons, reduced number of motor units, altered neuromuscular connectivity and selective denervation of type II fibers all lead to atrophy of fast-contracting type II fibers [75]. Contrarily, the fiber-type shift in patients with severe HF tends to occur before muscle atrophy, with a lower percentage of type I fibers and a higher percentage of type II fibers [76]. In addition, apoptosis of the skeletal muscle cells correlates with the severity of HF and is accompanied by decreased levels of the antiapoptotic factor B-cell lymphoma 2 (BCL-2) and increased levels of the pro-apoptotic factor BCL-2-associated X protein (BAX) [77].

### 4.6 Others

Aging is strongly associated with impairments in cardiovascular function, muscle strength and cognitive performance. Mechanistically, aging- associated mitochondrial dysfunction, decreased levels of protein synthesis and peroxisome proliferator-activated receptor γ coactivator 1-α (PGC1-α), as well as protein degradation, muscle atrophy and denervation, and a shift in energy metabolism to anaerobic metabolism all increase the risk of cardiomyopathy and other cardiovascular complications [78, 79].

In patients with HF, decreased skeletal muscle blood flow due to reduced cardiac output affects the oxygen supply to that organ, which leads to decreased muscle function. The skeletal muscle is an important organ of the body involved in glucose metabolism, and reduced muscle mass affects glucose homeostasis in the body. Several studies have revealed a relationship between blood glucose and depressive symptoms [80, 81].

In addition, visceral fat accumulation is a risk factor for chronic degenerative diseases, such as cardiovascular disease, dementia and depression. Another morphological aspect of aging skeletal muscle is the infiltration of fat into muscle tissue components. Fat can be contained in both adipocytes and deposited in muscle fibers, which is an important factor contributing to reduced blood flow and decreased muscle mass [82]. Patients with sarcopenia may be involved in muscle loss due to increased adipose tissue through increased proinflammatory cytokines and decreased release of muscle factors, leading to the development of cardiovascular disease [83].

## 5. Treatment

**Fig. 2.**
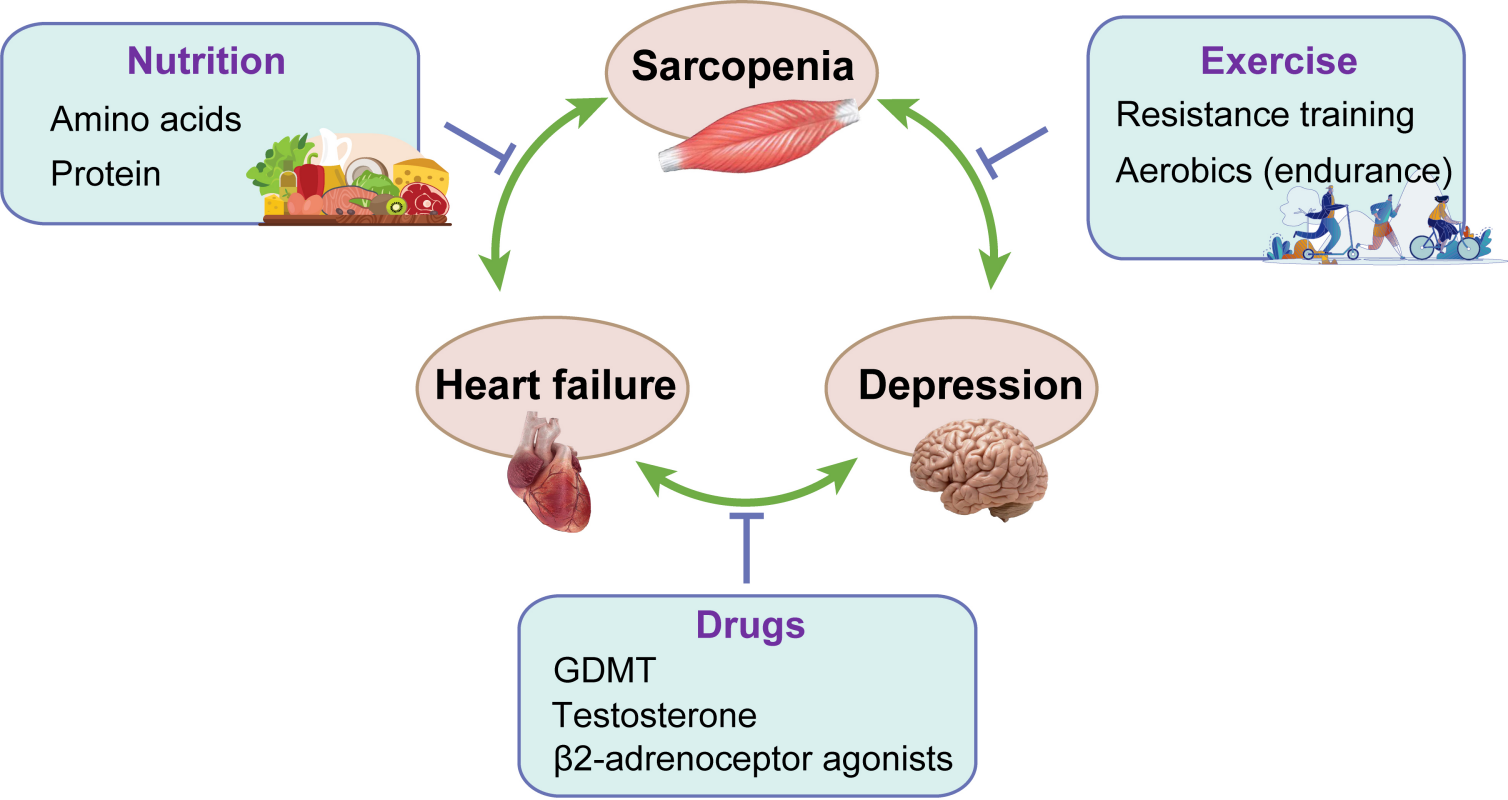
**The treatment of sarcopenia, HF and depression**. GDMT, guideline-directed medical therapy.

As to clinical studies on the pharmacological treatment of HF combined with depression, sertraline and paroxetine reduced depressive symptoms with fewer side effects. However, significant difference hadn’t been observed between sertraline and control group. Nonetheless, related studies revealed that sertraline and escitalopram did not achieve significant efficacy over placebo in the treatment of depression and HF [2]. Contrarily, the drug sacubitril/valsartan for HF improved depressive symptoms in patients with heart failure with reduced ejection fraction and depression [84]. The aforementioned studies suggest that antidepressant treatment in patients with HF and comorbid depression may improve their depressive symptoms but not their HF symptoms and prognosis and that effective HF treatment may improve the comorbid depressive symptoms. Thus, the treatment modality of HF with comorbid depression still needs to be explored, and probably, HF treatment should be the main focus. However, HF and body wasting should be detected early in the process of muscle loss in patients. The loss of body mass often begins with the loss of functional muscle. Although it is still not too late to start treatment in patients who have already experienced weight loss, an important therapeutic window might have been missed. It is important to first define the drug regimen for patients with HF, including maximum tolerated doses of angiotensin converting enzyme inhibitors (ACEI), β-blockers and mineralocorticoid-receptor antagonists (MRA), as described in the ESC [2] and ACC/AHA [1] guidelines. In addition to their beneficial effects on survival, some of these drugs have been shown to directly affect body weight. Other treatments include nutritional supplementation and increased physical activity. The treatment of sarcopenia, HF and depression is summarized in Fig. 2.

### 5.1 Nutrition

Both HF and sarcopenia are associated with anorexia, and patients with both conditions have low scores in nutritional assessment, suggesting that patients with HF and sarcopenia are more predisposed to malnutrition [85]. In addition, although nutritional assessment is currently performed less frequently in patients with depression, the pathogenesis of nutritional status of patients with depression remains unclear [28]. Similarly, anorexia nervosa is very common in patients with cachexia. Although cachexia cannot be reversed by improving nutritional intake, the beneficial effects may be achieved by increasing calorie, protein, or amino acid intake [14]. Saitoh *et al*. [86] have showed that Inflammation, use of loop diuretics, and cachexia are associated with an increased likelihood of anorexia in patients with HF, and patients with anorexia showed impaired functional capacity and poor outcomes. Some scholars have suggested that all patients with HF should take micronutrient supplements and avoid excess salt (≤6 g/day) and fluids (≤2 L/day) [14]. However, a recently published article showed no effect of dietary intervention to reduce salt intake [87]. Thus, the current recommendation of avoiding excess salt needs to be critically proofed and clinical randomized trials are needed further. While a high-calorie, protein-rich nutritional structure has shown benefit in cachectic patients, essential amino acids (especially branched-chain amino acids) may be more beneficial to patients with sarcopenia. Cheese, whey protein, leucine, and vitamin D have been studied and shown to improve muscle mass in the elderly [14, 88, 89].

#### 5.1.1 Amino Acids

Although the level of evidence for amino acid supplementation still falls short of clinical guidelines, there may also be some beneficial effects from nutritional intake of certain proportions of essential amino acids. Among them, branched-chain amino acids isoleucine, leucine and valine may be the major amino acid components of the skeletal muscle. Oral supplementation with these amino acids may enhance protein synthesis and inhibit protein hydrolysis in the muscle tissue, an effect that is most pronounced with leucine. Mechanistically, leucine promotes insulin signaling and glucose uptake in the skeletal muscle *via* the PI3K-AKT-mTOR pathway [90]. A double-blind, randomized, controlled study that included 38 patients with HF revealed improvements in peak oxygen consumption and 6-min walk distance after 2 months of oral administration of an essential amino acid blend [91]. Similarly, another double-blind, randomized, controlled trial revealed a slight improvement in peak oxygen consumption in elderly patients with chronic HF on oral supplementation with amino acids for 30 days [92].

#### 5.1.2 Proteins

A large part of the muscle mass is determined by protein synthesis and catabolism. The recommended intake of protein, in general, is 0.8 g/kg per day, and in patients with both HF and sarcopenia, a higher intake may be required [14]. Studies have demonstrated that after 6 weeks of high protein intake (20 g/day) and high-calorie (600 kcal) supplementation in patients with sarcopenia, these patients exhibited decreased plasma TNF levels and improved weight and quality of life [93]. Observational studies on sarcopenia support 1.0–1.2 g/kg protein per day as the optimal dietary protein intake to prevent sarcopenia [94].

### 5.2 Exercise

Exercise training is a nonpharmacological, low-cost, effective and safe treatment that is capable of improving depressive symptoms regardless of the patient’s psychiatric diagnosis, which makes it an excellent treatment strategy for depression [95]. Aerobic exercise activates peroxisome proliferator-activated PGC1-α, which induces mitochondrial biogenesis and inhibits the formation of FOXO3, a key protein for proteolysis [96]. In addition, anaerobic exercise also increases IGF-1 production by stimulating the PI3K/AKT pathway to stimulate myogenic fibronectin production [97]. The above processes further enhance protein synthesis and inhibits FOXO3 through AKT-mediated phosphorylation, thereby delaying sarcopenia [97]. Although a study assessing the effect of regular physical activity on local inflammatory parameters in skeletal muscle in patients with chronic heart failure (CHF) showed that aerobic training did not alter serum TNF, IL-1β, or IL-6 levels [98], it significantly reduced the levels of these cytokines and nitric oxide synthase in the skeletal muscle. Notably, aerobic training resulted in a 33% reduction in TRIM63 expression in patients with HF aged <55 years. This result was even more pronounced in the HF population aged >65 years (37% reduction in TRIM63 expression), suggesting that exercise training blocks the activation of UPS in patients with HF independently of age [99]. Meanwhile, patients with HF who underwent 12 weeks of exercise training exhibited a 36% reduction in local myostatin mRNA expression levels, although the serum myostatin expression was not altered, demonstrating the powerful therapeutic effect of exercise on sarcopenia [100].

Regular and appropriate physical activity can promote physical and mental health. As demonstrated in the HF-ACTION study, exercise can moderately reduce depressive symptoms in patients with HF [101]. However, excessive exercise can lead to emotional instability, reduce the body’s immunity, and affect physical health [102, 103]. Regular aerobic exercise that causes mild or moderate shortness of breath is recommended for patients in the ESC guidelines (Class I, Level A evidence) [2]. Similarly, regular physical activity is recommended in the ACC/AHA guidelines as a safe and effective way of improving body function (Class I, Level A evidence) [1]. Resistance exercise training may be a good strategy to improve the muscle structure in patients with sarcopenia. A randomized controlled trial revealed that after a 10-week period of high-intensity resistance training performed three times a week, patients exhibited an increase in muscle strength and endurance [104]. Also, Tieland *et al*. [105] found that protein supplementation combined with resistance exercise training for 24 weeks resulted in significant improvements in muscle mass and strength, suggesting that nutritional supplementation combined with exercise therapy may be more efficient.

### 5.3 Medications

A large number of medications may be beneficial to patients with HF and wasting. Potential drugs include anabolic steroids, anti-myostatin antibodies and ghrelin receptor agonists (such as anamorelin), anti-inflammatory drugs, appetite enhancers, proteasome inhibitors, and beta-adrenergic agonists.

#### 5.3.1 Guideline-Directed Medical Therapy (GDMT)

GDMT offers a beneficial role in combating physical wasting in addition to treating HF. For example, ACEI can improve mitochondrial function, increase IGF-1 levels, promote skeletal muscle glucose uptake and help treat sarcopenia [106]. Data from the SOLVD trial comparing the ACEI enalapril treatment group with the placebo group revealed that patients receiving enalapril had a 6% lower risk of weight loss than those receiving placebo [31]. In addition, in a small study of the β-blockers carvedilol and metoprolol in 27 patients with HF with or without cachexia, patients with cachexia (n = 13) gained a mean weight of 5.2 ± 9.6 kg after 6 months of β-blocker treatment, whereas those without cachexia gained only 0.8 ± 8 kg during the same period [107], demonstrating the benefit of β-blockers in the treatment of HF combined with cachexia. Contrarily, salt corticosteroid receptor antagonists, such as spironolactone, may assist in the treatment of sarcopenia in patients with HF by improving vascular endothelial function, reducing skeletal muscle apoptosis and enhancing muscle contractility [108].

#### 5.3.2 Testosterone

In addition to being an anabolic steroid, testosterone is a vasodilator in the coronary vasculature and pulmonary vasculature. Low testosterone levels are common in patients with HF and exacerbate cardiac dysfunction by altering peripheral vascular resistance, increasing cardiac afterload, and decreasing cardiac output [109, 110]. Decreased testosterone levels are also associated with decreased muscle mass and functional impairment [111]. In contrast, in patients with HF, low levels of testosterone are independently associated with exercise intolerance [112]. A study evaluating testosterone therapy in patients with HF revealed that testosterone therapy was associated with a relative increase in cardiac output and a decrease in systemic vascular resistance [110], with significant improvements in the Minnesota Living with HF Questionnaire scores in the testosterone-treated group. There was also a significant improvement in walking distance and grip strength in the testosterone-treated group [113].

#### 5.3.3 β2-Adrenoceptor Agonists

Several studies have demonstrated that β2-adrenoceptor agonists have been successfully used to either attenuate or reverse the loss of skeletal muscle mass induced by different experimental settings [114, 115]. The mechanism is that β2-agonists can increase protein synthesis and decrease protein catabolism, thereby increasing muscle fibronectin content and exerting a beneficial effect on muscle tissue [116]. Furthermore, a study in stable patients with chronic HF selected a long-acting β2-adrenoceptor agonist, clenbuterol, as an intervention and showed a significant increase in lean muscle mass and lean muscle/fat ratio in the treatment group after 12 weeks of administration. Clenbuterol has been widely used in athletes to improve athletic performance and has been shown to improve skeletal muscle recovery after orthopedic surgery. In cardiac studies, the use of high-dose clenbuterol during left ventricular assist device (LVAD) support has been shown to promote cardiac recovery. Although, clenbuterol was well tolerated by stable chronic HF patients from the results of this study, patients had a significantly shorter exercise duration after clenbuterol treatment [117]. Despite the use of the drug promotes an increase in muscle mass, it does not necessarily translate to an increase in muscle strength, and only the latter is considered to be associated with patient quality of life and long-term prognosis. Given that β2-Adrenocepter agonists are not recommended by HF guidelines and do not improve endurance in patients with stable chronic HF, caution is needed in the use of β2-Adrenocepter agonists in the HF patients. This study concludes that although the use of the drug promotes an increase in muscle mass, it does not necessarily translate to an increase in muscle strength, and only the latter is considered to be associated with patient quality of life and long-term prognosis. Given the limited effect of the drug on the skeletal muscles, it should be used with caution in patients with chronic HF.

## 6. Conclusions

Sarcopenia can occur at any stage of HF with comorbid depression, and it is not only a complication of HF and depression but also a risk factor for both. Mechanistically, there are many similarities and potential links between the pathogenesis of all three. In terms of treatment, the current therapy is focused on the treatment of HF, supplemented by the prevention and treatment of sarcopenia. Given that studies on amino acids and proteins are small studies, supplementations of amino acids and proteins are not part of the recommended therapies for these patients. Thus, larger randomised studies are still needed in the future. Because of the multiple pathogeneses involved, a single treatment cannot meet the clinical needs of the patients. As such, a combination of modalities is required, and the treatment is based on increasing physical activity and supplementation [118]. Improved nutritional status and exercise tolerance in patients with HF have the potential to improve HF and its comorbid depressive symptoms. Enhancement of the muscle function improves the prognosis of patients with HF and depression. The 2021 ESC guidelines for HF also recommend that all patients with chronic HF who are moderately active participate in exercise to improve their quality of life and reduce the hospitalization rate. For patients with severe comorbidities or frailty, supervised exercise may be considered as the basis for a cardiac rehabilitation program [2]. In addition, it is important not to neglect the issue of patient education to improve patient compliance. Furthermore, aside from making patients aware of the complexity of the disease, it is important to popularize multiple treatment modalities and lifestyles, such as supplementation with multiple nutrients along with reasonable physical exercise. Given the complex mechanisms of HF combined with depression and sarcopenia, more clinical studies involving all three diseases are needed in the future to gain a deeper understanding of the relationship between the three diseases and to optimize treatment.
